# Evaluation of a high throughput multiparallel stirred bioreactor system using an apple cell line

**DOI:** 10.1038/s41598-026-50148-3

**Published:** 2026-04-27

**Authors:** Amanda Lillberg, Gea Guerriero, Emmanuelle Cocco, Samuel Jourdan, Kjell Sergeant, Jenny Renaut, Maria Pajumo, Amir Akhgari, Heiko Rischer, Sylvain Legay

**Affiliations:** 1 VTT Technical Research Centre of Finland, Ltd, P.O. Box 1000, Espoo, FI-02044 Finland; 2 Environmental Research and Innovation Department, Institute of Science and Technology, Hautcharage, Luxembourg

**Keywords:** Plant cell suspension culture, *Malus* x *domestica*, Bioreactors, Metabolomics, Triterpenes, Biological techniques, Biotechnology, Engineering, Plant sciences

## Abstract

Cultivating dedifferentiated plant cells in bioreactors offers a continuous, scalable, reproducible, and fully controlled platform for producing valuable secondary metabolites. However, performance is highly dependent on cultivation conditions. In this study, we investigated the impact of different bioreactor configurations on the growth and metabolite production of an apple plant cell suspension culture. Stirred-tank bioreactors (STRs) with different vessel geometries (flat and round-bottom) were evaluated at the multi-liter scale (2–5 L) and compared, for the first time with plant cells, with a high-throughput small volume parallel system (240 mL). Using these mini-bioreactors, we studied the effect of impeller type (Rushton, marine, elephant ear) and pO2 cascade controls on cell growth and triterpene production. Results revealed that triterpene content and biomass yield varied significantly across cultivation systems and that plant cells were highly sensitive to shear stress, with variation coefficient reaching 56.2%-63.5%. Configurations with elephant ear-type impellers seemed to reduce shear stress, supporting stable triterpene accumulation (14.15 mg/g cells DW) and higher biomass (11.8 g DW/L). The high-throughput system closely mirrored multi-liter STR performance, demonstrating consistent and reliable prediction at larger scales. In contrast, wave bioreactors and flask cultures yielded distinct results, emphasizing the importance of continuous monitoring and system-specific optimization. Overall, these findings demonstrate that careful selection of bioreactor design and control strategy is critical for maximizing plant cell growth and metabolite production, and that small-scale high-throughput systems can be effectively used as process development tool for plant cell-based biomanufacturing.

**Supplementary Information:**

The online version contains supplementary material available at https://doi.org/10.1038/s41598-026-50148-3.

## Introduction

The cultivation of living cells to produce valuable metabolites and biomolecules has become a cornerstone of biotechnology. Among the diverse cell systems available, plant cell cultures (PCCs) have emerged as a unique platform with the potential to produce complex secondary metabolites, many of which have high pharmaceutical and industrial value^1^. In recent years, the PCC technology has experienced a resurgence of interest as a powerful alternative for producing plant secondary metabolites, high-value extracts and high-quality biomass^2^. PCCs are particularly advantageous for producing rare compounds that are scarce or challenging to obtain safely and sustainably from other plant biomass sources^3^. Additionally, PCCs enable the use of elicitation strategies to modify the chemical composition of specific plant genotypes, offering a significant advantage over conventional agricultural methods primarily under uncontrolled environmental conditions. Consequently, the technology has garnered considerable attention in the fields of natural products and alternative sustainable technologies^4,5^.

Dedifferentiated PCCs are derived from plant tissues and cultivated under controlled in vitro conditions. Plant cells possess unique biosynthetic pathways enabling specialized metabolite biosynthesis. However, PCCs require specific growth conditions, including optimal medium, environmental parameters and bioreactor design. This makes them more sensitive compared to microbial and mammalian cell cultures^6,7^.

In this paper, we investigated an apple cell line obtained from the ‘Reinette grise du Canada’ heirloom russeted variety^8^, aiming to produce increased amounts of pentacyclic triterpenes compared to fruits. These compounds belong to a large and diverse class of naturally occurring organic compounds composed of six isoprene units, resulting in a C30 structure. They are synthesized via the mevalonate (MVA) and/or 2-*C*-methyl-D-erythritol 4-phosphate/1-deoxy-D-xylulose 5-phosphate (MEP/DOXP) pathways in plants and exhibit ecological functions, such as feeding deterrence^9^, and bioactive properties including anti-inflammatory, antimicrobial, anticancer, hepatoprotective, and antioxidant properties (WO2020161221A1)^10,11^. This explains their value for industrial applications in the field of pharmaceuticals, cosmetics, and nutraceuticals^10^.

Stirred tank reactors (STRs) and wave-tank reactors (WTRs) are widely used bioreactor platforms for PCCs because their hydrodynamic environments can be tuned to balance efficient mass transfer with the pronounced shear sensitivity of plant cells. In STRs, relatively short mixing times and well-characterized flow patterns enable homogeneous nutrient and dissolved O_2_ distributions, which is critical given the large cell aggregates and high O_2_ demand often encountered in PCCs. Studies on mixing time in multi-impeller STRs have shown that appropriate impeller configurations can significantly reduce macromixing times while avoiding compartmentalization, thereby limiting O_2_ gradients that are known to negatively affect secondary metabolite formation in plant cells^12^. O_2_ transfer in STRs is typically described by kLa, which can be predicted across scales demonstrating that appropriate kLa can be achieved at moderate power inputs, although local turbulent dissipation rates near impellers may still pose a risk of mechanical damage to fragile plant cells^13^. Despite this challenge, STRs have been successfully applied for the production of recombinant glycoproteins in *Nicotiana tabacum* cell suspensions, where controlled agitation and aeration ensured stable glycosylation profiles at laboratory scale^14^. In contrast, WTRs rely on wave-induced motion and free-surface renewal, generating longer mixing times than STRs but markedly lower average shear rates and energy dissipation, which are advantageous for shear-sensitive PCCs. Experimental and computational fluid dynamics studies have shown that kLa, in wave bioreactors, increases monotonically with rocking speed and angle, reaching values sufficient for aerobic plant cell growth while maintaining hydromechanical stresses well below damage thresholds^15,16^. These characteristics have enabled successful scale-up of plant cell cultures producing isoflavones in disposable wave-based systems up to 100 L, combining gentle mixing with adequate O_2_ transfer^17^. Moreover, recent reviews highlight that disposable rocking and wave bioreactors are increasingly favoured for PCCs due to their low shear environment, simplified scale-up based on mixing and Froude number criteria, and reduced operational complexity compared to classical STRs^18,19^.

The early stages and process optimization of a new plant cell line are labour-intensive and often inefficient, increasing development costs and impacting technological transfer and return on investment. Many PCC experiments in the scientific literature use flasks^20,21^, providing less relevant data to subsequent process optimization and industrial scale-up. These systems’ requirements in terms of minimal volumes (1 L to 4 L) make them time consuming to optimize. To expedite PCC process development and obtain scalable data, we explored, for the first time with dedifferentiated plant cells, single use low volume miniature-STRs combining the benefits of flasks and STRs. We compared different impeller setups with cultures grown in flasks and STRs with varying vessel geometries, evaluating performance based on biomass and secondary metabolite production. By comparing multi-liter scale data with results from the miniature-system, we demonstrate the miniature-bioreactor’s suitability for selecting bioprocess parameters that can be effectively translated to larger volumes. Ultimately, this research aims to provide a comprehensive understanding of how to cultivate PCCs for industrial applications in the most cost- and time-effective manner.

## Results and discussion

### Establishment of apple cell cultures

**Fig. 1:**
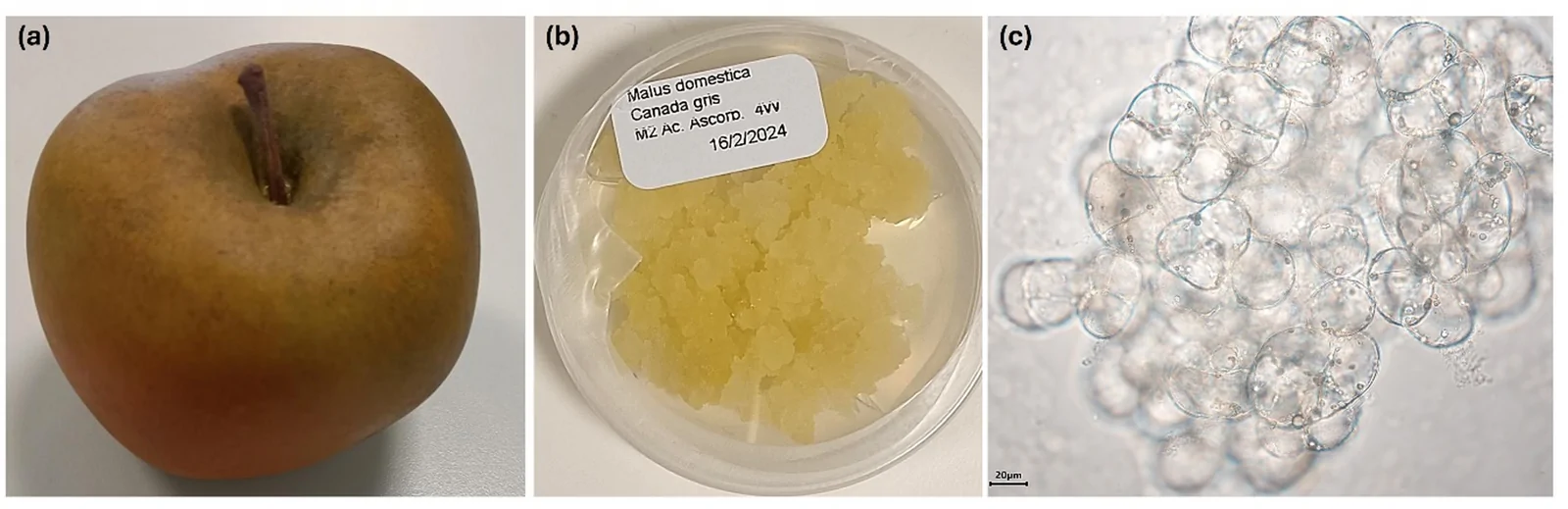
Sequential steps leading to the establishment of the *M.* x *domestica* cell suspension culture. (**a**): Fruit of *M.* x *domestica* “Reinette grise du Canada”, (**b**): callus grown on solid medium, (**c**): a cluster of cells grown as a suspension and observed with an optical microscope.

Friable and light yellow coloured calli were obtained from skin explants of the *M.* x *domestica* “Reinette grise du Canada” fruit (Fig. 1a-b); these calli were then used to establish cell suspension cultures. The suspensions showed the presence of a few small aggregates, typically within the millimeter range, which did not interfere with subsequent subculturing procedures using a pipette. Microscopically, the cell line consisted of uniformly shaped ovoid cells (Fig. 1c), frequently observed in small clusters. In flasks, the dry weight (DW) biomass yield was 11.1 ± 0.3 g/L.

The first part of this work consisted in the transfer of cell suspension cultures from flasks to multi-liter bioreactors. This transition in cultivation conditions is a critical phase in bioprocess development, as it introduces crucial changes in the environment that can impact plant cell physiology and productivity. Flasks often suffer from a lack of efficient mixing, causing concentration variances in culture conditions and leading to gradients in O_2_ and nutrients^22^. In contrast, dynamic systems such as STRs provide a better homogeneity of the culture, enhancing mass transfer and minimizing gradient formation. However, such improvement in process efficiency largely depends on adjusting suitable process parameters used (stirring speed, airflow rate, etc…), as well as on the cell line physiology and morphology. Homogeneity issues may finally arise again due to the larger volumes used at pilot and production-scale STRs^23^. Indeed, bioreactors can also develop gradients due to their size and complex hydrodynamics, which can lead to areas where cells have limited access to O_2_ or nutrients. Conversely, flasks usually offer smoother agitation leading to less shear stress compared to the impellers and aeration of a bioreactor.

Subsequently, the cultivation parameters and bioreactor configurations were evaluated using the high-throughput Ambr^®^ multi-parallel bioreactor system. The rationale was to use such a system to validate the bioprocess conditions established at the multi-liter scale for the cultivation of plant cell cultures. While these scale-down bioreactor models have been used for process development in bacteria^24^, yeast^25^ and mammalian cultures^26^, no studies have yet investigated their suitability for plant cell-based bioprocesses.

### Reference multi-liter apple cell cultivations

The selected multi-liter bioreactors included both STR and WTR (Supplementary Figure S1). Six different production platforms were assessed, with biomass production and secondary metabolite yield as key performance indicators.

When transferred to WTR and STR, a difference in the total dry biomass yield was observed, with higher values in WTR vessels (15.5 ± 1.1 g/L DW) compared to STR (13.2 ± 0.5 g/L DW, when averaging the DW values of all the units used). Such a difference may be due to the shear sensitivity of the studied apple cell line. Rocking motion of WTRs provides a low-shear environment that favors better viability and proliferation of shear-sensitive plant cells^27^. In contrast, while offering superior mixing and O_2_ transfer due to mechanical agitation and aeration via spargers, STR might generate higher shear forces that can impair the growth of sensitive cells. The uniform and mild mixing conditions in WTR create an optimal microenvironment for plant cells to thrive, potentially compensating the lower mixing and aeration typically associated with such reactor types^28^. Despite the advantages of WTR systems in providing a low-shear environment suitable for plant cells, their applicability for upscale production and development remains constrained by a lack of knowledge regarding upscaling parameters, such as the power input per volume (P/V) and the complexity to evaluate properly the Reynolds number (Re) in such systems (Table S5). These limitations complicate industrial scale-up efforts. Furthermore, despite providing the best biomass production results in the present work, we also observed a strong variability in phytochemical profiles in both untargeted and targeted analyses (see below), reducing the interest of using these WTR cultivations as low shear stress reference batches. Consequently, the WTR system was not further considered for scale comparison.

**Fig. 2:**
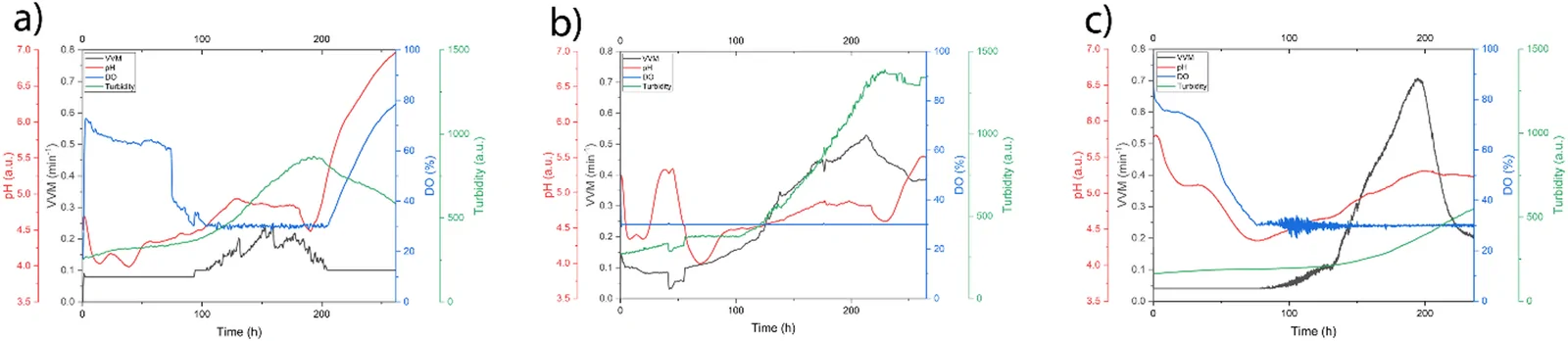
Profiles of VVM (black), pH (red), dissolved O_2_ (DO, blue) and turbidity (green) during the batch cultivation of *M.* x *domestica* cell suspensions in multi-liter stirred-tank bioreactor units (STRs). In **(a)** 2 L STR with round bottom; in **(b)** 2 L STR with flat bottom; in **(c)** 5 L STR with round bottom.

The cultivation of the cells in the different multi-liter bioreactors was characterized by differences in the pO₂, pH, and aeration rate for vessel volume per minute (VVM) profiles, indicative of variations in cell metabolism, gas exchange and transfer dynamics (Fig. 2). However, biomass production in all multi-liter bioreactors was in a similar range despite the observed differences during cultivation. The flat-bottom bioreactor yielded 13.3 g/L DW, which was consistent with the values observed in other systems: 13.1 g/L DW for the 2 L bioreactor, 14.0 g/L DW for the 5 L bioreactor with an airflow cascade, and 12.5 g/L DW for the 5 L bioreactor with a stirring cascade.

At the early stage of batches, a slower decrease in O₂ was observed in round bottom reactors, compared to the flat-bottom design. A possible reason that could explain, at least in part, such behavior is associated with a more efficient O_2_ transfer rate (OTR) in round bottom reactors. The vessel shape (flat or round bottom) has a significant influence on this parameter. However, previous flow dynamics studies showed weaker mixing in round bottom reactors^29,30^ compared to flat-bottom bioreactors. The latter generate stronger vortices, better mixing and consequently better OTR. This is not in accordance with the present observations and suggests that other parameters might drastically influence OTR in these reactors. Indeed, vessel equipment has a massive impact on nutrient and O_2_ management in bioreactors. OTR is also influenced by multiple parameters including T, mass transfer coefficient (K and L), and the gas/liquid interfacial area (a). These parameters are, in turn, affected by operational and design factors including agitation rate, sparger design which determine bubble size, bubble residence and, finally, the type and size of the impellers (Table S5). Consequently, these differences in O_2_ kinetics are multifactorial making it difficult to assess such different bioreactor architectures and brands. Another explanation might be associated with a reduced metabolic activity occurring at the early stage of these batches or a lower mixing occurring in round-bottom reactors. However, as the nutrient content and O_2_ levels are particularly elevated at the beginning of the batches, we are not considering this explanation as viable.

The pH graphs of the cell cultures collected during the growth in the different units showed similar features, albeit with either more or less pronounced shifts. The pH curves showed an initial drop, followed by a peak (at ca. 24 h) that was more marked in the flat-bottom 2 L bioreactor. The values then increased steadily until the end of the batches. This similarity across scales suggests that physiology is maintained despite the difference in reactor volumes, indicating that the behavior of the apple cells in larger reactors mirrors that at smaller scale.

### Utilization of a high throughput bioreactor system for the apple cell culture

The multi-parallel high-throughput Ambr^®^ bioreactor system was used to systemically assess the effect of bioreactor geometry on cultivation parameters within a single comparative platform. The use of a scale-down process in bioreactors offers significant advantages in streamlining the identification of critical parameters for cell cultivation during technology transfer to larger volumes^24^. Indeed, by working at more manageable scales, automated miniature bioreactor systems lower the risks, timelines, and costs associated with large-scale bioreactor runs by enabling the optimization of critical factors such as mixing, aeration, temperature, and pH.

Three different types of impellers, i.e., Rushton-, marine- and elephant ear-type, were evaluated for *M.* x *domestica* cultivation (*n* = 3, Supplementary Figure S1c– e, Table S5). Two cascade systems established in multi-liter bioreactors were adapted to a smaller scale. One setup adjusted the stirring speed in relation to the dissolved O_2_ (pO_2_) setpoint, a method widely used in microbial cultivations^31^. The other system adjusted the airflow rate based on the pO_2_ setpoint, a gentler approach commonly applied to shear-sensitive mammalian cells^32^. Both cascade setups were evaluated using vessels equipped with elephant ear impellers. A homogenous seed culture was used as inoculum for all multi-parallel high-throughput bioreactors.

The profiles obtained from vessels equipped with the elephant ear-type impellers and regulated with the two different pO_2_ regulatory cascades showed significant differences when considering pH and backscatter data. The setup with a stirring speed cascade appeared to be more stable, showing a clear increase in turbidity over the duration of the batch. This was confirmed by the end of batch biomass measurement (see below).

**Fig. 3:**
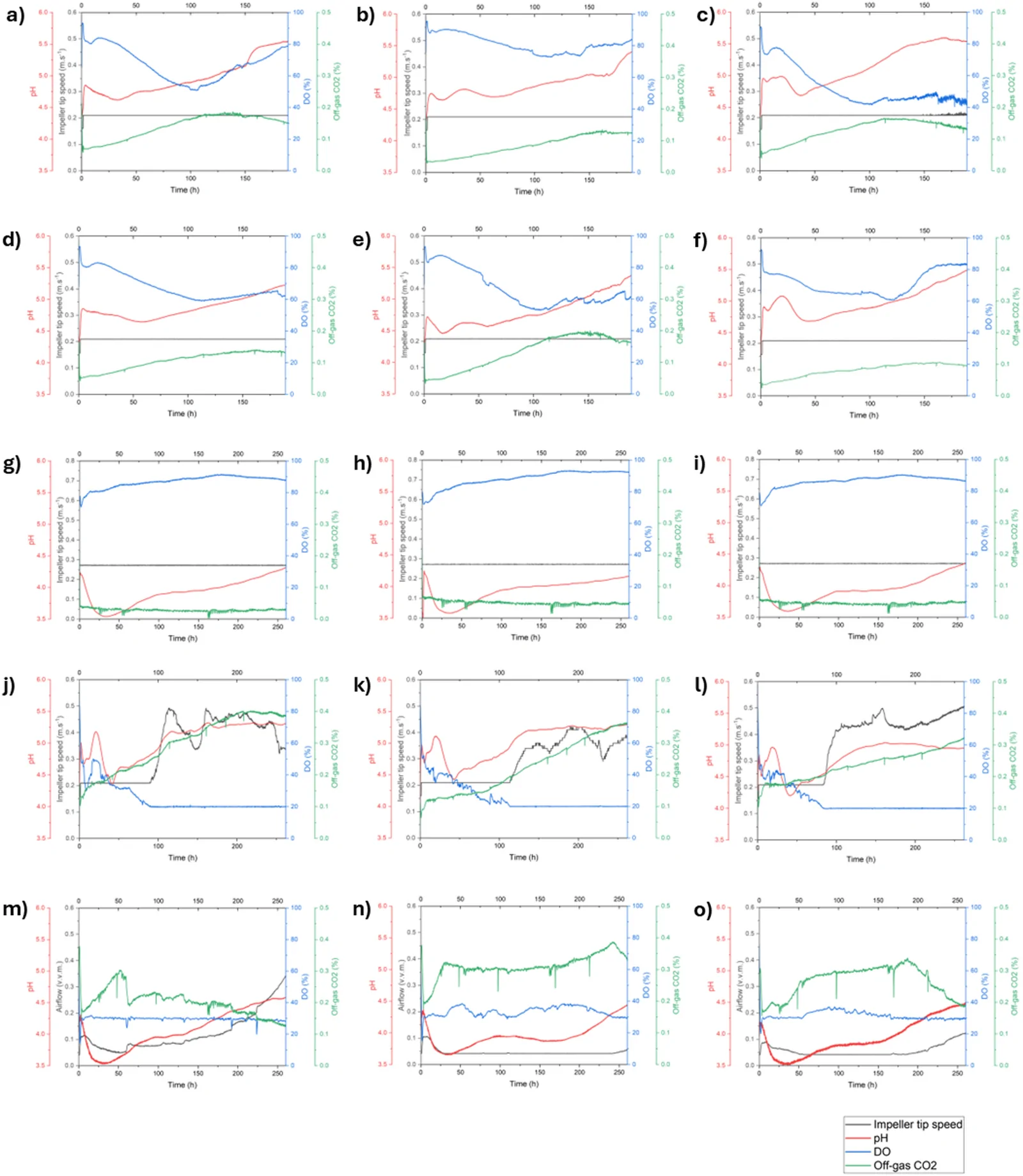
Profiles of impeller tip speed (black), pH (red), dissolved O_2_ (DO, blue) and CO_2_ evolution (off-gas, green) during the batch cultivation of *M.* x *domestica* cell suspensions in Ambr^®^ bioreactor units. In (**a-c**) Rushton impellers with pO_2_ setpoint at 20%; (**d-f**) Rushton impellers with pO_2_ setpoint at 30%; g-i marine impellers with pO_2_ setpoint at 20%; (**j-l**) elephant ear impellers with pO_2_ setpoint at 20%; (**m-o**) elephant ear impellers with pO_2_ setpoint at 30%. A stirring cascade control was used in the vessels (**a-l**), whereas an airflow cascade was applied in the vessels m-o.

Further comparisons of the pO_2_ level during cultivation between vessels equipped with Rushton and elephant ear impellers revealed a more rapid decrease in pO_2_ in the elephant ear vessels. The observation highlights significant differences in O_2_ transfer dynamics, resulting from the unique flow patterns, O_2_ distribution and mixing efficiency associated with each impeller type (Fig. 3, Table S5). This variation among reactors may result in reduced nutrient availability and lower O_2_ demand, in vessels with Rushton-type impellers, as previously observed^33^. These findings emphasize the critical role of determining the correct bioreactor configuration for plant cell lines with specific O_2_ demands while also considering their sensitivity to shear stress.

The miniature bioreactors demonstrated pH and pO₂ patterns similar to those of the multi-liter bioreactors equipped with elephant ear-type impellers. In most of the miniature bioreactors, the pH curve declined during the first 24 h before gradually increasing (Fig. 3) similarly to the multi-liter scale. The pO_2_ curve in vessels equipped with elephant ear-type impellers and airflow cascade regulation resembled the O_2_ demand observed in the 2 L STR with a flat bottom. These similarities suggest that miniature bioreactors can effectively replicate key physiological conditions observed in larger-scale systems. The pH and O_2_ transfer dynamics in the miniature bioreactor systems indicates their potential suitability for apple cell culture, providing a scalable and efficient model for further optimization and experimentation.

The PCA plots of the dissolved O_2_ (DO), pH and off-gas values collected from the Ambr^®^ miniature-bioreactors further illustrate the impact of impeller type, cascade mode, and pO₂ setpoint (Supplementary Figure S2, Table S5). DO values from miniature-bioreactors equipped with Rushton-type impellers displayed a scattered distribution, while those of marine-type operating in stirring cascade mode formed a distinct and tightly clustered group, separating them from elephant ear impellers, regardless of pO₂ setpoint or cascade mode. The separation of elephant ear impellers from the marine types, irrespective of cascade mode or pO₂ setpoint, suggests that their mixing characteristics lead to a different O₂ distribution pattern. The scattered distribution of Rushton-type impellers suggests higher variability in O_2_ transfer, likely due to the radial (e.g., horizontal) flow pattern splitting into two streams, one upwards and one downwards^34^, which creates stagnant regions near the vessel’s walls^35^ and results in less uniform O₂ availability along the vertical axis of the bioreactor. In contrast, the marine-type, as well as the elephant ear impellers in stirring or airflow cascade modes formed distinct and tightly clustered groups, suggesting a more uniform O₂ transfer. Such impellers create top-to-bottom rings, improving vertical mixing.

The pH values revealed a grouping of elephant ear impellers at 30% pO₂ setpoint in airflow cascade mode together with marine types at 20% pO₂ in stirring cascade mode. The elephant ear impellers at 20% pO₂ in stirring cascade also revealed a well-defined cluster, whereas the Rushton-type impellers showed a more dispersed pattern. The scattered pattern of Rushton-type impellers suggests that they provide less stable pH regulation, likely due to O₂ fluctuations impacting cell metabolism. In terms of off-gas values, the groupings were less distinct, with only marine-type impellers at 20% pO₂ in stirring cascade forming a tighter cluster.

#### *Biomass production and sugar consumption in high throughput bioreactor system*

The biomass yield results indicate that controlling the pO_2_ setpoint via stirring leads to a significantly higher biomass production (11.8 ± 1.9 g/L DW) compared with airflow cascade control (4.4 ± 0.8 g/L DW). The increased agitation probably enhances O_2_ transfer and mixing efficiency, thereby avoiding the stress associated with high airflow rates. In contrast, biomass production in vessels equipped with other types of impellers was comparatively lower, with Rushton impellers yielding 4 ± 0.4 g/L DW and 4.5 ± 1.4 g/L DW; and marine impellers achieving 3.1 ± 0.4 g/L DW (Supplementary Table S2). These results highlight the fact that vessels equipped with marine and Rushton impellers are inappropriate for such scale down system. These differences might result from variations in flow dynamics and mixing patterns compared to those occurring in larger reactors (5 L STR, ~ 13 g DW/L), highlighting the crucial impact of shear stress and O_2_ distribution, in these small reactors. It is also noteworthy that inoculum measurements between multi-liter reactors and high-throughput bioreactors might be slightly different, as the dry biomass was not measured in the present experiments. Nevertheless, we consider that the massive differences observed in this work cannot be only explained by such small initial variations.

To complement physiological and biomass data, the sugar consumption was measured (Supplementary Table S3). Vessels equipped with elephant ear- and marine-type impellers demonstrated an efficient sucrose hydrolysis compared to reactors equipped with Rushton impellers. However, only vessels equipped with elephant ear-type impellers and regulated by stirring cascade showed a significant decrease in simple sugars (glucose and fructose), compared to all other systems. This suggests that optimized metabolic conditions might be driven by stable O_2_ transfer and predominantly by moderate shear stress. In contrast, cultivations utilizing marine-type impellers exhibiting a relatively low O_2_ consumption, displayed a lower sugar consumption, as fructose and glucose levels remained at higher levels during the course of the batch with a slight decrease at the end of cultivation.

Reactors operated with Rushton-type impellers also displayed a low O_2_ consumption with a slight decrease in pO2 during the first 100 h follow by a stabilization of this parameter. Interestingly, and compared to other setups, sucrose hydrolysis remained incomplete until the end of the batches, suggesting a limited metabolic activity. This was confirmed by the simple sugar content, which increased over the course of the batch, indicating poor uptake compared with the elephant ear–type impeller/stirring speed cascade setup. This putative decrease in metabolic activity was further supported by the slowdown in O_2_ consumption observed after 100 h of batch duration. Theoretical Kla values ranged from 0.00085898s^− 1^ to 0.00866118 s^− 1^ which is comparable to previous work and to values obtained from multi liter reactors (Supplementary Table 5). Reynolds numbers calculated for each multi-parallel reactor setup revealed homogeneous results (49 > Re > 293) suggesting that laminar to weak transitional flows occurred in these reactors (Supplementary Table 5). For these reasons, we suspect that a possible explanation of the weak performance of vessels equipped with Rushton and marine impellers might be caused by shear stress, especially considering these reactors were equipped with two impellers compared to elephant ear impellers. However, further experiments are required to confirm this hypothesis.

#### Targeted triterpene analysis of apple cell cultivations

**Fig. 4:**
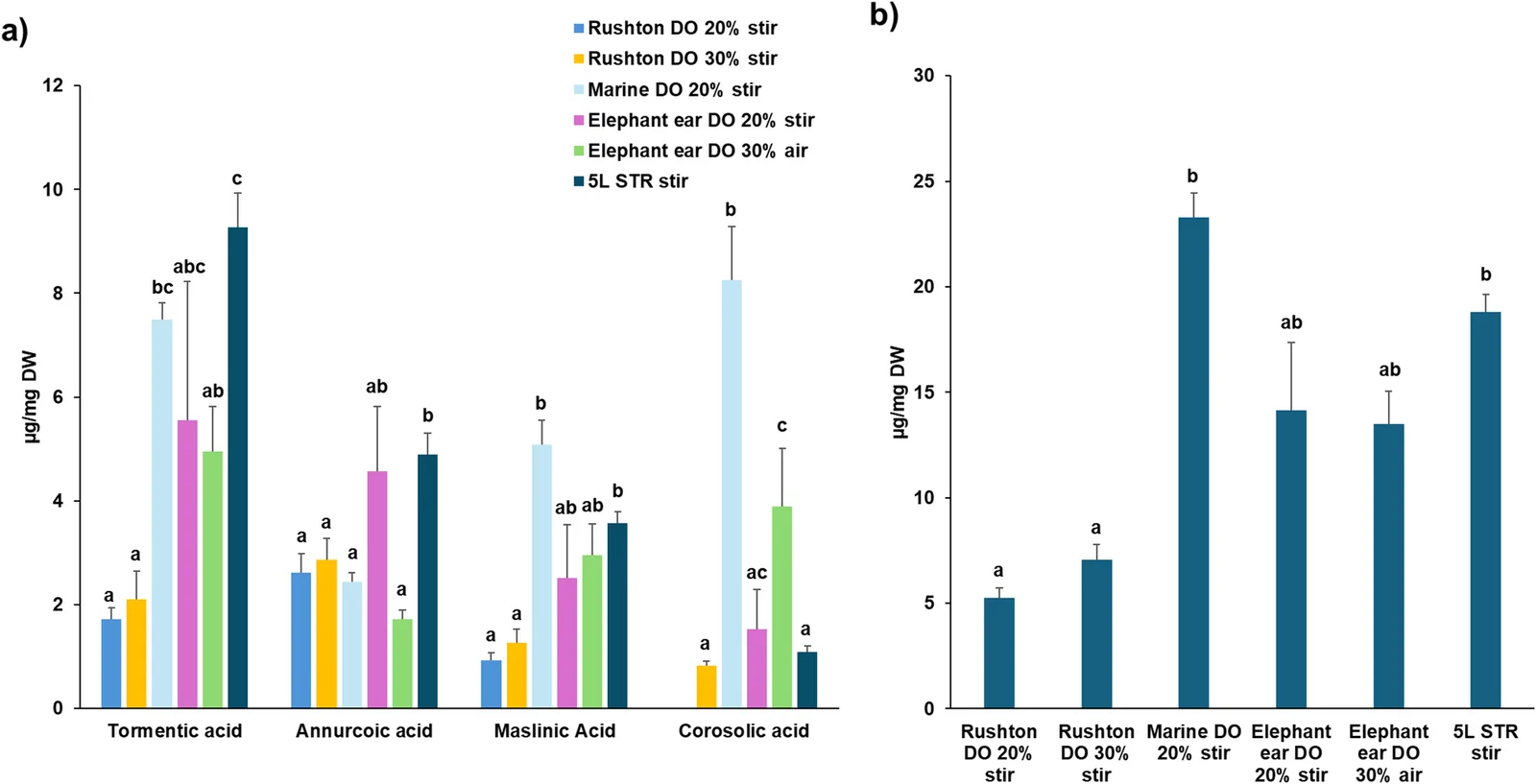
Pentacyclic triterpene content of **(a)** tormentic acid, annurcoic acid, maslinic acid, corosolic acid and **(b)** total triterpene content in *M.* x *domestica* cell suspensions cultivated in Ambr^®^ bioreactor units and 5 L STR. The error bar is the standard deviation of three biological replicates. The values are expressed as µg/mg dry weight of cells. Statistically significant differences between each compound are indicated with varying letters a – c (ANOVA, *p* ≤ 0.05).

Two distinct trends were observed in triterpene accumulation across different bioreactor configurations and oxygenation conditions. First, an overall lower triterpene content was found in the Ambr^®^ units with Rushton-type impellers under a stirring cascade (Fig. 4). Second, higher triterpene concentrations were detected in the Ambr^®^ miniature bioreactors with elephant ear-type impellers with both cascade settings, in vessels equipped with marine impellers, and in the 5 L STR. This separation was particularly evident for tormentic and maslinic acids, suggesting that the accumulation of these compounds is more responsive to dynamic flow conditions occurring during batches.

The triterpene profile in the apple cell suspension culture reflected the composition of pentacyclic triterpenes in apple fruits, as it was predominantly governed by the ursane, oleanane and lupane series, respectively deriving from α-amyrin, β-amyrin, and lupeol, which are further oxidized by a cytochrome P450 belonging to the family of CYP716A175^36^. These compounds are mostly found in the waxy coating of leaves and of fruits such as apples and pears^37^. In apple fruits, pentacyclic triterpenes represent 32 to 70% of the epicuticular waxes and their total amount can reach up to 60 mg per apple fruit (about 3 to 4 mg/g DW). Furthermore, it has been shown that the pentacyclic triterpene composition in apple is also altered by the fruit skin phenotype^38^. Indeed, while waxy apple fruits display ursane and oleanane series’ pentacyclic triterpenes, russeted apple displayed a decreased accumulation of these triterpene series and an increased accumulation of the triterpenes from the lupane series^39^. Finally, russeted apples, such as the ‘Reinette grise du Canada’, display a significant accumulation of triterpene hydroxycinnamate esters, mostly betulinic acid-3-*trans*-caffeate^8,39^. The major triterpenic acids in the current cell line were tormentic, annurcoic, corosolic, and maslinic acids, while no triterpene hydroxycinnamate esters were observed (Fig. 4). It is noteworthy that all these compounds belong exclusively to the ursane and oleanane series. Previous work performed on a dedifferentiated ‘Cox Orange Pippin’ apple cell line showed a similar alteration of the triterpene composition compared to those observed in fruits but with a massive accumulation of 3-*O*-p-coumaroyl tormentic acid (WO2020161221A1)^38,40^.

These results suggest that hydrodynamic forces influence triterpene production in apple cell cultures. The lower accumulation of triterpene in the Ambr^®^ miniature-bioreactor equipped with Rushton-type impellers may be, again, due to suboptimal shear forces, which could limit biosynthetic activity^41^. The highest triterpene accumulation was observed in vessels with marine-type impellers, which reduced the shear stress and maintained stable O_2_ distribution. However, in these batches, the global triterpene profile was significantly altered compared to 5 L STR with a relative higher amount in corosolic acid (precursor of tormentic acid) and a lower accumulation in annurcoic acid. Vessels equipped with elephant ear-type impellers showed slightly lower total triterpene contents but displayed a global profile similar to those observed in the multi-liter STR (Fig. 4b). Finally, it is noteworthy that WTRs, despite providing good results related to biomass yields, displayed inconsistent results regarding the triterpene contents. Indeed, among the three conducted batches, only one showed measurable triterpene content (above detection limits) with total triterpenes reaching 12.8 ± 0.8 mg/g DW. The quantification of triterpenes further highlighted the differences between STR and WTR systems. While STRs were characterized by distinct metabolomic patterns depending on impeller type and cultivation conditions, WTRs showed considerable variation across cultivations, which did not align with the most prominent cultivation outcomes observed in STRs (Supplementary Figure S3). Previously WTRs were mainly used to produce plant cell biomass due to their cost efficiency and effective production scale^42,43^. However, to our knowledge, batch-to-batch variation and reproducibility of target compound production have not been previously evaluated. The quantification of triterpenes revealed substantial variations as evidenced from the total amount of triterpenes extracted from cells cultivated in WTRs (see below, supplementary Figure S3). In contrast, laboratory-scale and smaller-scale STRs exhibited distinct clusters, indicating more reproducible cultivation outcomes.

### Untargeted metabolomics for evaluating bioreactor systems

**Fig. 5:**
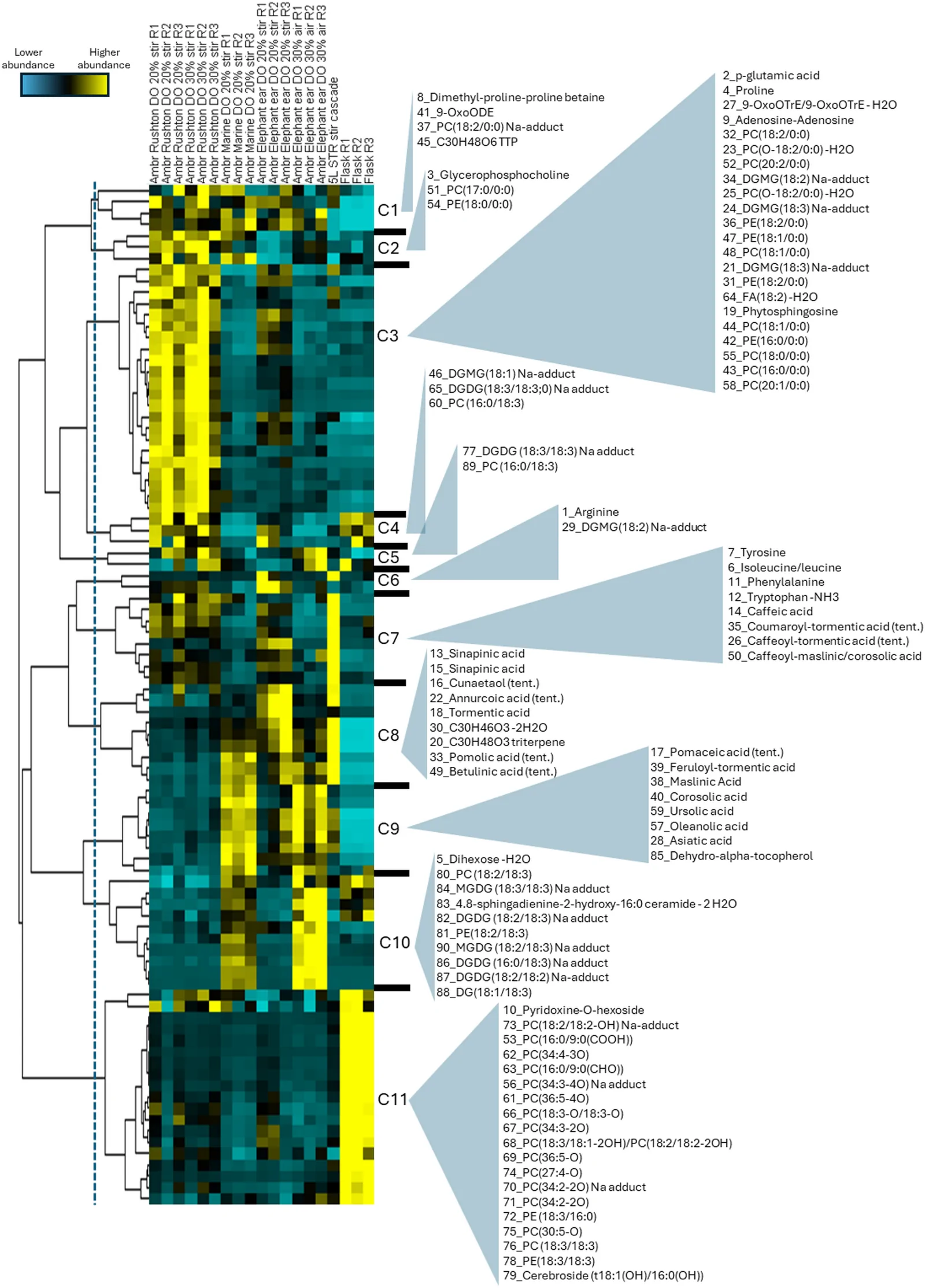
Hierarchical clustering performed on compounds displaying significant differences in accumulation (ANOVA, *p* < 0.05, *n* = 3, metabolomic data obtained from UPLC-tripleTOF analysis, Supplementary Table S4) using uncentered Pearson correlation and a complete linkage agglomeration method. Clusters were defined using a minimum Pearson correlation of 0.50.

To gain a deeper understanding into how different bioreactor configurations influence the secondary metabolism of apple cell cultures, untargeted metabolomics was performed. The analysis revealed distinct patterns of metabolite accumulation across different reactor systems (Supplementary Figure S4), clearly distinguishing samples based on bioreactor geometry and cultivation conditions. As evidenced by the hierarchical clustering, the metabolite abundances resulted in eleven major clusters (Fig. 5). Notably, clusters 1 to 3 were characterized by a pronounced accumulation of various lysophospholipids (LPLs), particularly phosphatidylcholine PC(20:0/0:0), PC(18:2/0:0), PC(17:0/0:0), PC(20:1/0:0), phosphatidylethanolamines PE(18:0/0:0), PE(18:2/0:0), PE(16:0/0:0), as well as monoacylglycerols, such as diacylglycerol-derived monoglycerides DGMG(18:2), DGMD(18:3). These clusters also included osmoprotectants and amino acid-related metabolites, such as dimethyl proline-proline betaine, proline, oxo-octadecadienoic acid (oxoODE), and glutamic acid, and were predominantly enriched in miniature-reactors equipped with Rushton-type impellers. These findings suggest that higher shear stress, or O_2_ limitation (caused by poor mixing), triggered by the use of Rushton impellers, might induce stress responses which, in turn, trigger the increased abundance of LPLs. In this respect, it is interesting to note that LPLs can be produced by the action of phospholipases^44^. Furthermore, prior research on *Taxus cuspidata* cell cultures has demonstrated the activation of phospholipase D and C in response to mechanical shear stress, suggesting a mechanosensitive regulatory mechanism in lipid signaling pathways^45^. Future research could explore whether phospholipase activation is a consistent feature in plant cells grown in bioreactors equipped with Rushton-type impellers or other conditions imposing shear stress. However, this hypothesis must be assessed in further shear stress experiments.

Cluster 4, while still showing enrichment in lipid species such as digalactosyldiacylglycerol (DGDG (18:3/18:3) and PC (16:0/18:3), was observed not only in the Rushton-equipped miniature-reactors but also in shake flasks, indicating that certain lipid biosynthetic pathways may be upregulated under more heterogeneous culture conditions. In contrast, cluster 7 exhibited a different signature, with higher abundance of aromatic and branched-chain amino acids, including tyrosine, isoleucine/leucine, and phenylalanine, alongside caffeic acid and triterpene derivatives. This profile was particularly associated with multi-liter STR (and to a lesser extent with Rushton- and elephant ear-equipped miniature-reactors with pO_2_ setpoint under stirring cascade), suggesting that specific hydrodynamic conditions in these systems may promote the synthesis of these compounds.

Cluster 8 further supported this trend, showing higher levels of phenylpropanoid compounds and triterpenes, such as sinapic acid, cunaetol, pomolic, annurcoic, and betulinic acids, with enrichment observed in both multi-liter STR and mini-reactors equipped with marine or elephant ear-type impellers.

Cluster 9 was marked by a higher accumulation of different pentacyclic triterpenes, namely pomaceic acid, feruloyl-tormentic acid, maslinic, corosolic, ursolic, oleanolic, and asiatic acids in miniature-reactors with marine- and elephant-type impellers and in the multi-liter STR. This cluster clearly indicates that triterpene production in cells grown in miniature-reactors with marine or elephant ear-type impellers closely mirrors the performance typically observed in multi-liter STR with same impeller type.

Clusters 10 and 11 predominantly featured membrane lipid species, with cluster 10 enriched in polyunsaturated lipids, such as PC (18:2/18:3), digalactosyldiacylglycerol DGDG (16:0/18:3), DGDG(18:2/18:2), DGDG(18:2/18:3), diacylglycerol DG(18:1/18:3), and cluster 11 characterized by a predominance of PC with long chains, including PC(36:5), PC(27:4), PC(30:5), PC(34:3), PC(34:2), PC(34:3). The acyl chain length was shown to be associated with lower membrane fluidity^46^, suggesting that under the shear stress imposed by impellers, cells may increase the incorporation of polyunsaturated lipids to enhance membrane fluidity as an adaptive response.

## Materials and methods

### Callogenesis and establishment of the cell suspension culture

Apple fruits (*Malus x domestica* var. ‘Reinette grise du Canada’) were sampled during spring-autumn 2013 at the Walloon Agronomic Research Center in Gembloux (CRAW, Gembloux, Belgium), as described^8^. Permissions from CRAW and complete sourcing documents have been obtained and stored. The fruits were quickly rinsed with tap water and the skin was excised from a slice comprising also part of the parenchyma with a scalpel after having performed a sterilization step with 5% (v/v) bleach supplemented with 0.05% (v/v) Tween 20 for ten minutes under agitation (120 rpm), followed by three washes in sterile distilled water. The fruit skin was cut in small squares (ca. 0.5 cm x 0.5 cm) and incubated (with the parenchyma side facing upwards) on callus induction (CI) medium (Murashige & Skoog-MS basal salts and vitamins, supplemented with 2,4-D 1 mg/L, kinetin 0.2 mg/L sucrose 30 g/L, agar 8 g/L, pH 5.8) in darkness and room temperature (RT). Once vigorously growing calli appeared, they were maintained on modified Linsmaier & Skoog medium^47^(LS basal salts and vitamins with 2,4-D 1 mg/L, kinetin 1 mg/L, ascorbic acid 0.1 g/L, sucrose 30 g/L, agar 8 g/L, pH 5.8). Ascorbic acid was added to the medium to prevent calli from excessive browning. For the establishment of the cell suspension cultures, approximately 0.5 g calli were transferred to 25 mL of liquid medium and incubated at 26 °C, 120 rpm under darkness. The suspension was brought to 100 mL by the progressive addition of fresh medium (over a period of ca. 4 weeks). The cell suspension was sub-cultured every 2 weeks at a density of 25% (v/v).

### Cultivation in bioreactors

The apple cell line was grown using different bioreactor setups and scales. Cells were grown in two 2 L bioreactors with round bottom and flat bottom, respectively (Biostream, Doetinchem, Netherlands; Minifors 2™, Infors HT Bottmingen, Switzerland). In addition, two 5 L bioreactors with round bottom were utilized for the cultivation (Biostream, Doetinchem, Netherlands; BioFlo320, Eppendorf, Germany). Agitation was achieved using either two marine-type impellers placed at (i) the lower end of the stirring bar and (ii) at 2/3 height of the culture volume height or one elephant ear type impeller as described in Table 1. The impeller diameters were 45 mm and 55 mm for 2 L and 5 L bioreactors, respectively. Reactors were equipped with EASYFERM ARC (pH) and VISIFermDO ARC (DO) probes (Hamilton company, Reno, Nevada, US). The pH probes were calibrated using pH 4 and pH 7 buffers before sterilisation. The DO was calibrated after autoclaving and temperature stabilisation (26 °C) using a 2-point calibration (0 and 100%) setup using air flow in reactors set at 2 VVM and an impeller tip speed set at 1.15 m·s^− 1^. For 2 L and 5 L batches, a cell growth quantifier BioR™ sensor (SBI scientific bioprocessing, Baesweiler, Germany) was placed at the bottom (1/3 for vessel bottom) surface of the reactor vessel to measure turbidity (backscatter) along the course of the batch. These sensors provide qualitative but not quantitative data about the culture turbidity, biomass production and have to be considered as a trend indicator due the inherent aggregation of plant cells. The impeller tip speed was fixed and ranged from 0.37 m·s^− 1^ to 0.42 m·s^− 1^. pH was not regulated whereas DO was maintained at 20% or 30% of the maximum O_2_ saturation capacity using an airflow or stirring cascade as described in Table 1. Reactors were inoculated at 20% of the final culture volume (35 g fresh weight FW/L final after inoculation) using cells grown in flasks up to the late exponential phase stage (9 days culture). At the end of batches, the produced biomass was collected and filtered with a Büchner funnel equipped with a vacuum system using Miracloth (22–25 μm, Merck, Darmstadt, Germany). The biomass was further freeze-dried to measure the dry weight (DW).

For cultivation under varying conditions, the apple cell line was grown in a wave bioreactor (Biostat RM, Sartorius, Germany) equipped with a 10 L wave bag (CultiBag RM, Sartorius, Germany), with a final working volume of 4 L. The cultivation was initiated with an inoculation density of 35 g/L FW, and the corresponding parameters were set as detailed in Table 1.

Additionally, the Ambr^®^ 250 high-throughput, multi-parallel miniature-bioreactor system (Sartorius, Germany) was utilized for the apple cell suspension cultivation. A total of 15 vessels were operated with three different impeller types including Rushton, marine, and elephant ear, as detailed in Table 1. A batch mode cultivation was initiated with a working volume of 240 mL and an initiation density of 30 g/L (FW) for each vessel. Dissolved O_2_ (DO) levels at 20% and 30% were used as indication either for the stirring cascade or the airflow cascade activation. The ranges for the cascades were set as 0.2–0.5 m·s^− 1^ or 0.04–0.62 VVM for the stirring cascade and airflow cascade, respectively. The temperature was kept constant at 26 °C. The cultures were harvested after 9–11 days. The cells were filtered using vacuum filtration as described above.

**Table 1:** Parameters used for the cultivation of *M.* x *domestica* “Reinette grise du Canada” cell suspensions in different wave-tank bioreactor (WTR) and stirred-tank bioreactor (STR) units.

Bioreactor type	Bioreactor WV (L)	Cascade control	Stirring (rpm)	Airflow (VVM)	pO_2_ setpoint (%)	Impellers
WTR	4	**-**	22 (8–10° angle)	0.03	-	-
STR (Flat)	0.25	Stir	200–500*	0.2	20	2 Rushton
STR (Flat)	0.25	Stir	200–500*	0.2	30	2 Rushton
STR (Flat)	0.25	Stir	200–500*	0.2	20	2 marine
STR (Flat)	0.25	Stir	200–500*	0.2	20	1 elephant ear
STR (Flat)	0.25	Air	200	0.04–0.2*	30	1 elephant ear
STR (Flat)	2	Air	150	0.05–0.38*	30	2 marine
STR (Round)	5	Air	130	0.04–0.71*	30	2 marine
STR (Flat)	5	Stir	50–200*	0.2	20	1 elephant ear

*This parameter was automatically controlled by cascade based on the pO_2_ setpoint. The range indicates the lower and higher limits during the run.

### Untargeted metabolomics

Lyophilised cells were ground with a Retsch mill (Retsch, GmbH, Haan, Germany, 250 μm final fineness) and extracted in EtOH 70% (v/v) with a ratio 1:20 w/v. The slurry was agitated at RT for 10 min at 140 rpm, then passed through filter paper, and the filtrate evaporated to dryness with a rotary evaporator at 40 °C under vacuum. The dry extract was homogenised in a mortar with pestle, dissolved in 1 mL of MeOH 85% v/v and 0.1% of formic acid and passed through 0.22 μm PTFE syringe filter (Millex-LG, Merck KGaA, Darmstadt, Germany). Five µL were injected in a UPLC eLambda 800 nm system (Waters Corporation, Milford, MA, US). The separation was performed on a reverse-phase Acquity UPLC BEH C18 column (Waters Corporation, Milford, MA, US). The solvents used were (A) water + 0.1% FA and (B) acetonitrile-ACN + 0.1% FA. A solvent gradient was applied: 99% A 0–16 min, 95% A 16–35 min, 60% A 35–45 min, 100% B 45–50 min, 99% A 50–54 min, and 99% A 54–60 min, at a flow rate of 0.5 ml/min. The column was maintained at 50 °C during the whole run. Mass spectrometric analysis was performed with a TripleTOF 6600+ (Sciex, Framingham, MA, US) and a DuoSpray Ion Source operating in both negative and positive ion mode. LC-MS runs were uploaded in Progenesis QI (v: 2.3; Nonlinear Dynamics, Newcastle upon Tyne, UK) and compounds identified using the LIST workflow based in the in-house database^48^. Level 2 identifications were obtained by matching experimental MS/MS spectra with available resources and should be considered as tentative based on the available evidence. Standards for the commonly found triterpenes (mentioned below) allowed level 1 identifications for these compounds which can therefore be considered as identified^49,50^.

### Quantification of pentacyclic triterpenes

The protocol for sample preparation and HPLC analysis was adapted from the method previously described by Andre et al.^36^. In brief, an exact amount between 300 and 500 mg of lyophilized material ground with stainless beads was combined with 15–25 mL of absolute ethanol. The mixture was homogenized with a vortex for 10 s and a sonic bath for 10 min, then shaken for 1 h at room temperature. After centrifugation at 4,000 x g for 15 min, the supernatant was collected and filtered through a 0.20 μm filter (water-wettable PTFE) and subjected to HPLC analysis. Each sample was analysed in triplicate using a Waters Acquity UPLC system (Waters Corporation) coupled with a Diode Array Detector (UPLC-DAD). Separation was carried out on a reverse-phase Acquity UPLC BEH C18 column (2.1 × 100 mm, 1.7 μm particle size, Waters Corporation). The eluents used were 0.05% *ortho*-phosphoric acid in water (A) and 0.05% *ortho*-phosphoric acid in methanol (B), with the following gradient: 0 min, 75% B; 2 min, 75% B; 16 min, 82% B; 25 min, 100% B; 27 min, 100% B; 27.5 min, 75% B; 30 min, 75% B. The flow rate was set at 0.3 mL min^− 1^, and the column temperature was maintained at 40 °C. Tormentic acid, maslinic acid, corosolic acid and annurcoic acid were identified based on their retention times and spectral data compared with standards and quantified at 210 nm using five-point calibration curves within the concentration range of 2.5–100 µg mL^− 1^.

### Sugar analytics

The sugar consumption in bioreactor cultivations was analysed using a high-pressure liquid chromatography (HPLC) system, Alliance 2695 (Waters Corporation, US) as described in^42^. The detection range for analysed sugars was 0.1–6 g/L. All samples were diluted with 5 mM H_2_SO_4_ to a total volume of 1 mL for the analysis. The results were subsequently processed using Empower 3 software (Waters Corporation, US).

### Statistical methods

The results were presented as mean value of biological replicates (*n* = 3, if not otherwise stated), with standard deviation indicating the error and variation between the replicates. Statistical analyses for triterpene contents were performed with SPSS Statistics 28 software (International Business Machines Corp., US). The homogeneity of variances was tested using Levene test (*p* < 0.05). One-way ANOVA test was used together with Tukey’s HSD for datasets with homogenous variances; otherwise, Dunnett T3 was used. Analytics were conducted for confidence levels: *p* < 0.05, *p* < 0.01 and *p* < 0.001.

## Conclusions

The secondary metabolite production varied significantly across different cultivation systems explored in this study. Plant cells demonstrated a high sensitivity to shear stress, which altered their secondary metabolism. In this study, configurations utilizing marine- and elephant ear-type impellers emerged as superior for cultivating the apple cell line by reducing shear impact, thereby providing an environment that supports stable secondary metabolism. Additionally, the high-throughput cultivation approach closely mirrored the outcomes observed in STRs at the multi-liter scale, while demonstrating strong consistency and reliability in bioprocess adaptation. However, wave bioreactors and flask-based cultivation methods produced distinct outcomes compared to stirred systems, highlighting the significance of continuous process monitoring. These findings underscore the necessity of tailoring cultivation strategies to the specific requirements of each cultivation system.

## Supplementary Information

Below is the link to the electronic supplementary material.

## Data Availability

Data will be made available on request.
