# Supplementary material for: Evaluation of a high throughput multiparallel stirred bioreactor system using an apple cell line
Source: Sci Rep. 2026 Apr 27;16:19468. doi: 10.1038/s41598-026-50148-3 (PMC13287454; doi:10.1038/s41598-026-50148-3)
Supplement: Supplementary file 2 — Supplementary Material 2 [file 41598_2026_50148_MOESM2_ESM.docx]

**Supplementary material**

**Supplementary material includes Figures S1 – S4 and Tables S1 – S4.**

**
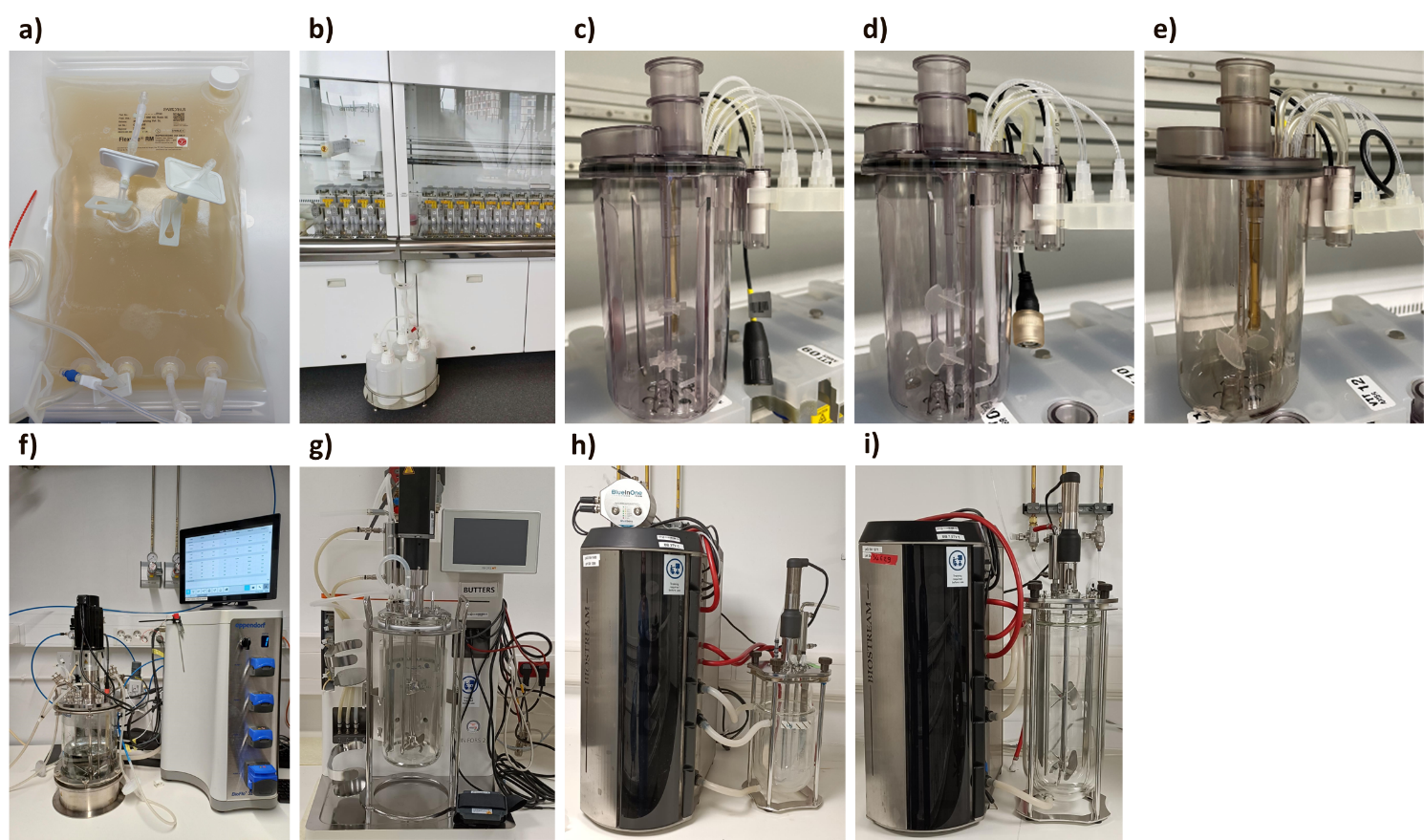
**

**Figure S1:** Bioreactor units used in the study. (a): Wave-tank reactor (4L WV), (b): overview of the Ambr^®^ 250 high-throughput multi-parallel bioreactor units, (c) Ambr^®^ unit (240 mL WV) with Rushton impellers, (d) Ambr^®^ unit (240 mL WV) with marine impellers, (e) Ambr^®^ unit (240 mL WV) with elephant ear impellers, (f) stirred-tank reactor (2L, WV) with flat bottom, (g) stirred-tank reactor (4L, WV) with round bottom, (h) stirred-tank reactor (2L, WV) with round bottom, (i) stirred-tank reactor (5L, WV) with round bottom.


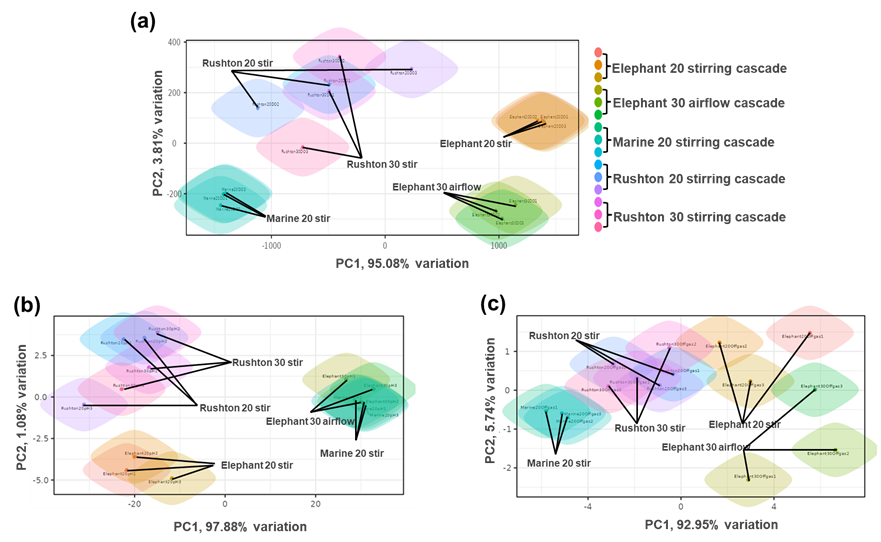


**Figure S2.** PCA plots of dissolved O_2_ (DO) (a), pH (b) and off-gas values (c) in Ambr^®^ mini-bioreactors with different impeller types, cascade modes and pO2 setpoints.


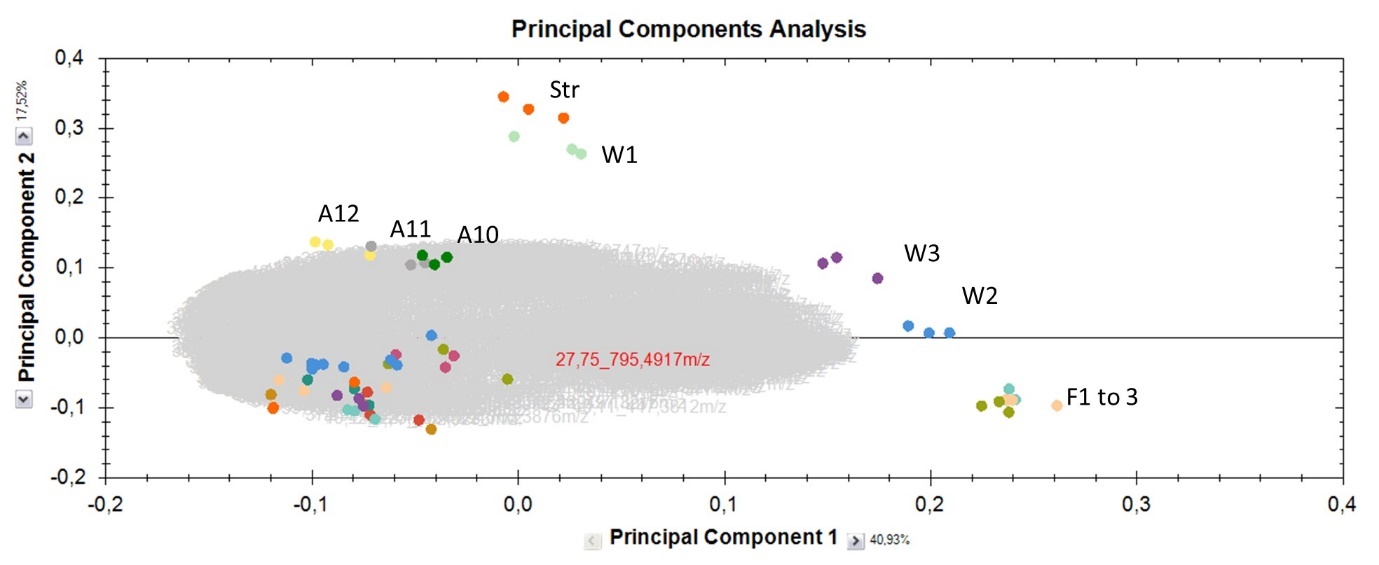


**Figure S3:** Principal component analysis (PCA) of the metabolomics datasets (1733 features) obtained for the cell suspensions grown in flasks (F), wave-tank reactors (W), stirred-tank reactor (Str) and Ambr^®^ bioreactor units (A).


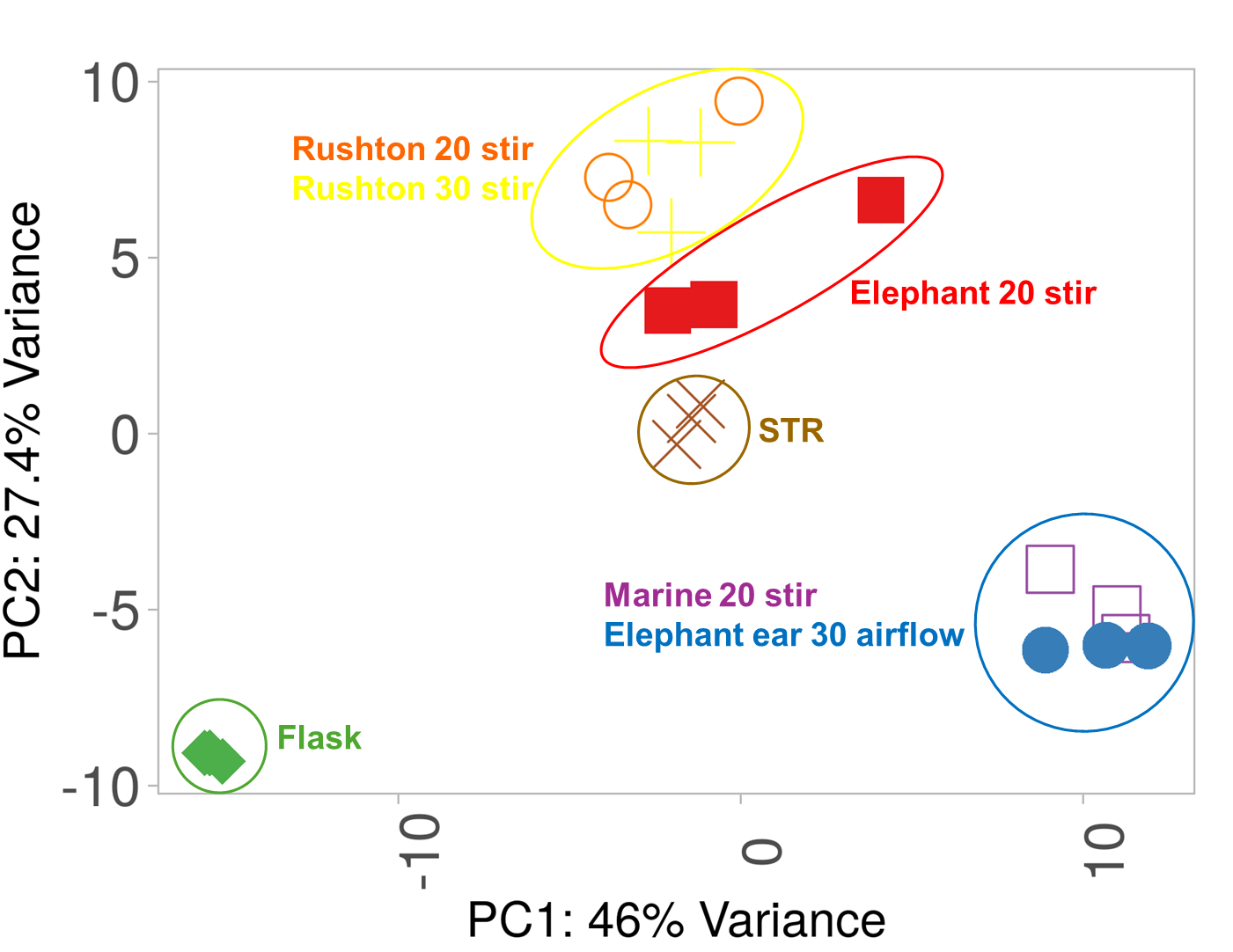


**Figure S4:** Principal component analysis (PCA) of the metabolomics data sets obtained from Ambr^®^ high-throughput miniature bioreactor system, flasks and 5 L STR.

**Supplementary Table S1:** Pentacyclic triterpene content in *M.* x *domestica* cell suspensions cultivated in 2 L STRs. The error is the standard deviation of three technical replicates. The values are expressed as µg/mg dry weight of cells.

| **Condition** | **Tormentic acid (µg/mg DW ± SD)** | | | **Annurcoic acid (µg/mg DW ± SD)** | | | **Maslinic acid (µg/mg DW ± SD)** | | | **Corosolic acid (µg/mg DW ± SD)** | | | **Total triterpene**  **(µg/mg DW ± SD)** | | |
| --- | --- | --- | --- | --- | --- | --- | --- | --- | --- | --- | --- | --- | --- | --- | --- |
| 2 L STR  DO 20% air | 24.3 | ± | 0.5 | 11.1 | ± | 0.4 | 6.1 | ± | 0.2 | 5.2 | ± | 0.1 | 46.7 | ± | 0.7 |
| 2 L STR  DO 30% air | 16.5 | ± | 1.2 | 14.4 | ± | 1.1 | 5.0 | ± | 0.5 | 3.1 | ± | 0.1 | 38.9 | ± | 1.7 |

**Supplementary Table S2:** Fresh and dry biomass (FW; DW; N=3) obtained when cultivating the *M. domestica* cell suspensions in flasks and different bioreactor units. The error is presented as a standard deviation (± SD) of three biological replicates and the statistically significant differences between each cultivation are indicated with varying letters A – C (ANOVA, p**≤**0.05).

| **Condition** | **FW (g/L)** | | | **ANOVA (p≤0.05)** | **DW (g/L)** | | | **ANOVA (p≤0.05)** |
| --- | --- | --- | --- | --- | --- | --- | --- | --- |
| Flask | 155.3 | ± | 1.8 | A | 11.1 | ± | 0.3 | B |
| WTR | 189.4 | ± | 37.8 | A | 15.5 | ± | 0.9 | A |
| Ambr® Rushton  DO 20% stir | 61.9 | ± | 14.9 | B | 4.5 | ± | 1.4 | C |
| Ambr® Rushton  DO 30% stir | 54.2 | ± | 7 | B | 4 | ± | 0.4 | C |
| Ambr® Marine  DO 20% stir | 37.7 | ± | 7.3 | B | 3.1 | ± | 0.4 | C |
| Ambr® Elephant ear DO 20% stir | 140.5 | ± | 30.1 | A | 11.8 | ± | 1.9 | AB |
| Ambr® Elephant ear DO 30% air | 59.4 | ± | 15.7 | B | 4.4 | ± | 0.8 | C |

**Supplementary Table S3:** Quantification of sugars during the cultivation of *M.* x *domestica* cell suspensions in Ambr^®^ bioreactor units: Rushton DO 20% stir cascade, Rushton 30% stir cascade, marine 20% stir cascade, elephant ear 30% air flow cascade and elephant ear 20% stir cascade. The error is presented as a standard deviation (± SD) of three biological replicates and the statistically significant differences between each vessel are indicated with varying letters A – J (ANOVA, p**≤**0.05) for each sugar concentration.

| **Condition** | **Time (h)** | **Sucrose** | | | **ANOVA** | **Glucose** | | | **ANOVA** | **Fructose** | | | **ANOVA** | **Total sugars** | | | **ANOVA** |
| --- | --- | --- | --- | --- | --- | --- | --- | --- | --- | --- | --- | --- | --- | --- | --- | --- | --- |
|  |  | **(g/L ± SD)** | | | **(p≤0.05)** | **(g/L ± SD)** | | | **(p≤0.05)** | **(g/L ± SD)** | | | **(p≤0.05)** | **(g/L ± SD)** | | | **(p≤0.05)** |
| Rushton  DO 20% stir | 1.4 | 19.5 | ± | 0 | A | 3.4 | ± | 0 | J | 3.2 | ± | 0 | GHI | 26.2 | ± | 0.1 | J |
|  | 41.8 | 16.5 | ± | 0.2 | B | 5.3 | ± | 0.5 | HIJ | 5.2 | ± | 0.5 | EFGH | 27.6 | ± | 1.2 | HIJ |
|  | 114.3 | 7.5 | ± | 1 | D | 9.5 | ± | 0.8 | EFG | 8.1 | ± | 0.5 | CD | 26.9 | ± | 1 | EFG |
|  | 160.8 | 2.3 | ± | 0.6 | F | 10.5 | ± | 0.4 | DEF | 8.4 | ± | 0.5 | CD | 22.2 | ± | 1.4 | DEF |
|  | 185.5 | 1 | ± | 0.4 | G | 10.7 | ± | 0.6 | DE | 8.3 | ± | 0.8 | CD | 20.9 | ± | 1.7 | DE |
| Rushton  DO 30% stir | 1.4 | 20.7 | ± | 0.5 | A | 3.2 | ± | 0.4 | J | 3 | ± | 0.4 | FGHI | 27.8 | ± | 0.9 | J |
|  | 41.8 | 17.2 | ± | 0.7 | B | 4.9 | ± | 0.4 | IJ | 4.8 | ± | 0.4 | EFGH | 28 | ± | 1.2 | IJ |
|  | 114.3 | 7.7 | ± | 1.1 | D | 10.2 | ± | 0.9 | DEF | 9 | ± | 0.9 | CD | 28.9 | ± | 1.1 | DEF |
|  | 160.8 | 1.9 | ± | 1.1 | FG | 11 | ± | 0.5 | CDE | 9.1 | ± | 0.8 | CD | 23.5 | ± | 0.4 | CDE |
|  | 185.5 | 0.6 | ± | 0.4 | FG | 10.3 | ± | 1.2 | DEF | 8.4 | ± | 1.3 | CD | 20.8 | ± | 2.1 | DEF |
| Marine DO 20% stir | 1.1 | 20.1 | ± | 1.2 | A | 4.8 | ± | 0.2 | IJ | 5.2 | ± | 0.1 | EFG | 30.2 | ± | 1.4 | IJ |
|  | 49.1 | 6.9 | ± | 0.4 | DE | 12.6 | ± | 0.5 | BCD | 12.9 | ± | 0.5 | B | 32.4 | ± | 0.9 | BCD |
|  | 97.1 | 0 | ± | 0 | G | 15.6 | ± | 0.6 | A | 16.2 | ± | 0.6 | A | 31.8 | ± | 1.2 | A |
|  | 169.1 | 0 | ± | 0 | G | 15 | ± | 0.5 | AB | 15.8 | ± | 0.1 | A | 30.8 | ± | 0.5 | AB |
|  | 217.1 | 0 | ± | 0 | G | 15.5 | ± | 0.2 | A | 16 | ± | 0 | A | 31.4 | ± | 0.2 | A |
|  | 265.1 | 0 | ± | 0 | G | 10 | ± | 0.4 | DEFG | 9.5 | ± | 0.3 | CD | 19.5 | ± | 0.3 | DEFG |
| Elephant ear DO 20% stir | 1.1 | 20.3 | ± | 0.1 | A | 2.9 | ± | 0 | J | 2.6 | ± | 0 | HI | 25.9 | ± | 0.1 | J |
|  | 41.8 | 12.9 | ± | 0.2 | C | 7.3 | ± | 0.6 | GHI | 7.1 | ± | 0.6 | DE | 28.2 | ± | 1.1 | GHI |
|  | 114.3 | 0 | ± | 0 | G | 12.6 | ± | 0.5 | BCD | 10.3 | ± | 0.6 | C | 23.3 | ± | 1.1 | BCD |
|  | 160.9 | 0 | ± | 0 | G | 10.5 | ± | 0.6 | DE | 7.3 | ± | 0.8 | DE | 18.5 | ± | 1.4 | DE |
|  | 208.3 | 0 | ± | 0 | G | 7.8 | ± | 1.1 | FGH | 4.3 | ± | 1.3 | FGH | 13.2 | ± | 2.4 | FGH |
|  | 263.3 | 0 | ± | 0 | G | 2.6 | ± | 2.1 | J | 1.1 | ± | 1.3 | I | 5.9 | ± | 3.4 | J |
| Elephant ear DO 30% air | 1.1 | 21.6 | ± | 1.2 | A | 5.2 | ± | 0.2 | HIJ | 5.5 | ± | 0.2 | EF | 32.3 | ± | 1.5 | HIJ |
|  | 49.1 | 4.9 | ± | 0.2 | E | 13.6 | ± | 0.8 | ABC | 14.2 | ± | 0.9 | AB | 32.6 | ± | 1.9 | ABC |
|  | 97.1 | 0 | ± | 0 | G | 15.1 | ± | 0.1 | AB | 15.7 | ± | 0.2 | A | 30.8 | ± | 0.2 | AB |
|  | 169.1 | 0 | ± | 0 | G | 14.5 | ± | 0.6 | AB | 14.7 | ± | 0.7 | AB | 29.2 | ± | 1.3 | AB |
|  | 217.1 | 0 | ± | 0 | G | 13.7 | ± | 0.6 | AB | 13.8 | ± | 0.7 | AB | 27.6 | ± | 1.3 | AB |
|  | 265.1 | 0 | ± | 0 | G | 10 | ± | 0.4 | DEFG | 14.6 | ± | 0.3 | AB | 24.5 | ± | 0.8 | DEFG |

**Supplementary Table S4:** [Supplementary Table S4.xlsx](https://vttgroup.sharepoint.com/:x:/r/sites/InnCoCellsVTT_LIST/Shared%20Documents/General/Apple/Manuscript/Submission%20folder/Supplementary%20Table%20S4.xlsx?d=w89705bbb724d4584a33e92027a902987&csf=1&web=1&e=YL9jDu)

**Supplementary Table S5:** Theorical kLA, P/V and Reynolds number calculated from available data. Kla was calculated using method and parameters described in Van Riet (1979) article (doi:10.1021/i260071a001). P/V data were calculated as described in Barradas et al. 2011 (doi: [10.1186/1753-6561-5-S8-P47](https://doi.org/10.1186/1753-6561-5-S8-P47)) using appropriate Np values (doi: 10.1002/elsc.201600096, <https://doi-org.proxy.bnl.lu/10.1016/B978-0-12-387785-7.00016-5>). Reynolds number was calculated using method and constants described in Manickavasagam et al. 2025 (doi: 10.1038/s41598-025-92385-y).

| **Setup name** | **Theoric kLa (s-1)** | **P/V (W/m3)** | **Reynolds Number** |
| --- | --- | --- | --- |
| Ambr Rushton (200rpm) | 0.00219 | 4.74 | 49.87 |
| Ambr Rushton (500rpm) | 0.00866 | 74.07 | 165.64 |
| Ambr Marine (200rpm) | 0.00149 | 2.20 | 88.30 |
| Ambr Marine (500rpm) | 0.00590 | 34.43 | 293.25 |
| Ambr Elephant ear (200rpm) | 0.00164 | 2.64 | 72.51 |
| Ambr Elephant ear (500rpm) | 0.00646 | 41.25 | 240.81 |
| Ambr Elephant ear (0.04 vvm) | 0.00086 | 2.64 | 72.51 |
| Ambr Elephant ear (0.2 vvm) | 0.00164 | 2.64 | 72.51 |
| Minifors 2L (Flat bottom) | 0.00271 | 11.80 | 203.59 |
| Minifors 2L (Flat bottom) | 0.00610 | 11.80 | 203.59 |
| Biostream 5L (Round bottom) | 0.00220 | 15.47 | 322.29 |
| Biostream 5L (Round bottom) | 0.00695 | 15.47 | 322.29 |
| BioFlo320 5L (Flat bottom) | 0.00071 | 0.45 | 101.31 |
| BioFlo320 5L (Flat bottom) | 0.00569 | 28.55 | 622.79 |
